# Writing Units or Decades First in Two Digit Numbers Dictation Tasks: The Case of Arabic—An Inverted Language

**DOI:** 10.3390/brainsci11111518

**Published:** 2021-11-16

**Authors:** Deia Ganayim, Ann Dowker

**Affiliations:** 1 Special Education Department, Al-Qasemi Academic College of Education, Baqa El-Garbiah 3010000, Israel; 2 The Arab Center for Mind, Brain and Behavior (ACMBB), Sakhnin 3081000, Israel; 3 Learning Disabilities Department, The College of Sakhnin for Teacher Education, Sakhnin 3081000, Israel; 4 Educational Counselling Department, The Max Stern Yezreel Valley Academic College, Yezreel Valley 1930600, Israel; 5 Department of Experimental Psychology, University of Oxford, Oxford OX2 6GG, UK; ann.dowker@psy.ox.ac.uk

**Keywords:** two-digit numbers, transcoding, Arabic, dictation, numerical development, counting systems

## Abstract

This study investigated the effect of educational level and of the syntactic representation of numbers in Arabic on the task of transcoding two-digit numbers from dictation. The participants were primary, junior-high, and high school pupils and higher education students. All spoke Arabic as a mother tongue. They performed a transcoding task, namely writing two-digit numbers from dictation. Units first\decades first writing patterns were collected depending on the differential syntactic structures of the two-digit number dictated (decades first: whole tens; units first: teen numbers; identical units and decades, remaining two-digit numbers). The findings reveal that in general, Arabic speakers adopt a decades-first writing pattern for two-digit numbers, especially when it is consistent with the syntactic structure of two-digit numbers, as in whole-tens numbers. This decade-first writing pattern is more evident and consistent in junior-high school, high school, and higher education than in primary school due to the improvement in mathematical skills and second and third languages. However, this pattern is modulated by the syntactic complexity of the unit–decade structure. This complexity is more pronounced in two-digit numbers whose processing is more dependent on numerical syntax. Thus, whole-tens numbers, teen numbers, and identical-decade–unit numbers are less complex than the remaining two-digit numbers.

## 1. Introduction

Numbers may be presented in different ways; for example, Arabic numerals (01234567893) are distinct from number words (e.g., one, two, three), which themselves vary by language. These diverse representations evoke various mental processes that are involved in the understanding, production and calculation of Arabic numerals and verbal numbers (number words) [1].

All educated adults can shift from one notational system (number symbol) to another without substantial difficulty. They can write an Arabic numeral according to dictation or read an Arabic numeral aloud; for example, 4 is equal to ארבע in Hebrew, أربعة in Arabic, and four in English. Transcoding is the process in which data or information is decoded into another format such as video transcoding and linguistics transcoding. This step of translating numbers from one context to another is called “transcoding” or “conversion” and is considered a basic aspect of the development of number processing.

Transcoding is involved in many daily activities, such as specifying the time, reading a price, or registering telephone numbers, and it is also a prerequisite for arithmetical calculations. However, transcoding processes are not as easy as they might initially seem to be. The difficulty of transcoding is apparent in children, who require several years of practice to acquire the relevant skills [2,3,4,5,6,7,8,9]. It also occurs as a deficit in some adults with brain damage [10,11,12,13].

Children learn the verbal number system long before they learn to read or write Arabic numerals, also called Indo-Arabic Numerals, and this acquisition order seems to impact the transcoding process. According to the Sapir–Whorf hypothesis, language influences mechanisms of thinking). Few people nowadays accept the strong Whorfian hypothesis that language completely determines and constrains thought; but there is significant evidence for a weaker version, whereby language has some influence on certain aspects of cognition, including mechanisms of number processing [14,15], The structure of the verbal counting system appears to affect the difficulty of understanding numbers and using them in mathematical operations. Thus, the linguistic structure of number words, such as the order of units and decades in two-digit numbers, may influence how numbers are processed in tasks, such as in deciding which number is larger [16]. Therefore, recent studies of number processing have centered on how and to what degree language affects number processing [17,18].

There are many types of numerical systems around the world with different internal structures. They vary both in their lexical structure, i.e., the system of words used to represent numbers (Arabic: واحد٬ إثنان٬ ثلاثة; Hebrew: אחד, שתיים, שלש; English: one, two, three), and in their syntactic structure, i.e., how individual lexical units are composed in order to generate a larger verbal number [19]. In addition to the verbal number systems, there are digit number systems that also vary in their internal structure. They differ in their syntactic structure, or the order of units, decades, and hundreds, etc. Thus, the transcoding of numbers from one system (verbal or digits) to another requires control of the coding mechanisms of these verbal and digit systems. Bilinguals and multilinguals must control these transcoding processes in more than one language, which increases the complexity for them, especially when the transcoding processes differ between their languages.

The purpose of the present study is to study Arabic speakers’ transcoding from the verbal system to the digits system, in Arabic. It investigates this topic with regard to the numerical system of the Arabic language, which has several distinctive characteristics. In particular, this numerical system includes somewhat unusual feature: writing numbers in a different direction (left to right) from writing text (right to left in Arabic) and similarly presenting tens and units in different orders in the spoken and written number systems (the inversion feature).

### 1.1. Cross-Linguistic Studies of Transcoding

Different languages have different transcoding systems for numbers. Studies on transcoding between languages have provided significant insights into how language influences the acquisition of number transcoding. Although researchers have examined the transcoding system in various European and East Asian languages, very few have explored transcoding in the Arabic language. The existing research does suggest that language influences the nature and difficulty of transcoding and indicates the importance of studying transcoding in a wider variety of languages [20]. An important early study of transcoding in different languages was carried out testing French and Belgian children, who had different systems of writing numbers from dictation [8]. For example, in French, 70 and 90 are written as a complex “60 + 10,” “soixante-dix,” and “(4 × 20) + 10” “quatre-vingt-dix”. In Belgium, they are more transparent: “septante” and “nonante.” However, 80 is read as “4 × 20” or “quatre-vingt” in the two countries. The study found that French children made significantly more transcoding errors. Notably, these composite structures were often not combined lexically. On the contrary, the errors often mistook the partial lexicalization of each of the elements that comprise the decade; for example, writing the number eighty-two as 4202, 422, or 802. These errors demonstrate the strong influence of language on the acquisition of a digit number system. The task was completed through transcoding tasks that investigated the possible confounding effect of the understanding of verbal and written numbers. The results indicate that errors in transcoding tasks were significantly more likely, which was due to errors concerning the relation between the word number and digit rather than errors in reading comprehension.

Another previous study compared French- and English-speaking fifth grade (10-year-old) childrens’ performance in number transcoding [21]. Whereas English two-digit number names follow the decimal structure (base 10), the structure of French two-digit number words over 60 follow a vigesimal structure (base 20). Children undertook two number transcoding tasks. While children were generally successful at the tasks, English-speaking children significantly outperformed French-speaking children for numbers following a vigesimal structure in French compared to a decimal structure in English (i.e., numbers > 60). These findings show that verbal number name structures influence children’s performance in numerical tasks, even though fifth-grade children have well passed the initial stage of acquiring transcoding skills for two-digit numbers. These findings highlight the importance of language specificities in number transcoding.

To date, no systematic developmental study has been published on transcoding in Arabic. However, there have been several studies of transcoding by children speaking European languages with the inversion feature, such as German and Dutch. However, in a comparison of the transcoding performance of children who speak German and French in Austria and Belgium, the error rate was higher among German-speaking children, and their types of errors were clearly related to the inversion feature in German for two-digit numbers. Additionally, German children needed more time to learn to write two-digit numbers correctly from dictation. The researchers discovered that German children have developed a unique strategy to overcome this problem: reversing the digit numbers in writing from right to left [22]. Transcoding is affected not only by general rules but also by linguistic characteristics [23,24]. In the German language, where the unit digit precedes the decade digit, more substitution errors have been found [24,25,26]. In contrast, fewer substitution errors were found in Japanese since its number system is highly transparent compared to that of German [27]. In a transcoding study in the Czech language, in which numbers can be represented as either “units-decades” or “decades-units”, this order of number words affected the number transcoding errors [23]. These findings clearly indicate that specific linguistic structures, such as the complex structure of two-digit numbers in French and the inversion feature for two-digit numbers in German, can influence number transcoding.

Child speakers of Dutch (an inverted number language) and French (a non-inverted number language) were asked to write Arabic digits to dictation [28]. They were also given tests of language and working memory. Although the number of change errors (e.g., hearing 46 but writing 56) was equal in both groups, the number of substitution errors (e.g., hearing 46 but writing 64) was significantly higher in Dutch-speaking than in French-speaking children. Regression analyses confirmed that language was the only significant predictor of substitution errors. By contrast, aspects of working memory components, in contrast, were the only significant predictors of change errors.

The interaction between transcoding, math performance and working memory ability was analyzed in a large sample of over 25,000 Dutch children, from kindergarten to the end of primary school, who responded to transcoding items using a computer adaptive system [29]. Inversion errors declined with age but did not disappear completely, even for those in the final year of primary school.

The writing of two-digit numbers in 5–7-year-old English- and German-speaking children was investigated during their first year of formal education [30]. They were interested in the influence of number word inversion at the item level on number writing. As in previous studies, they found that German-speaking children made more inversion errors for numbers larger than 20 than English-speaking children. Though English-speaking children were less likely than inversion errors in English-speaking children. These errors occurred more often for number words that are inverted at the item level, i.e., teen numbers. Thus, inversion errors are more common for inverted spoken number words, even in a language where most number words are not inverted.

### 1.2. Transcoding and Models of Number Processing

One aim of the present study was to investigate whether the participants’ performance was compatible with any or all of several models of transcoding and number processing, which had been developed with regard to speakers of European languages. These models are listed in Table 1 and will be briefly described here. The triple code model was proposed which posits that transcoding employs auditory-verbal representation [31]. This model includes a direct route between different numerical representations, thus, verbal and Arabic numerals can activate phonological representations of the words directly without the semantic mediation or indirectly through semantic mediation [31,32,33].

The abstract modular model was proposed, which consists of two systems, one of number production and the other of comprehension (verbal format and numerical format), in addition to the central abstract representation of numbers, wherein the quantity is represented by a set of positions and values consisting of the exponents of 10 [34]. According to this model, the first step in producing a verbal number is the creation of a syntactic pattern of the number from its semantic representation.

The encoding complex model was proposed [35,36], whereby specific codes of the format and modality represent the numbers (see Table 1). The existence of intermediate representations was proposed (IR) [37] that relate to the lexical representations of numbers and their numeric representation (see Table 1). For example, 24 activates the IR of (4 + 20, four-and-twenty) in Arabic and (20 + 4, twenty-four) in English and Hebrew.

On the basis of previous studies on number transcoding by Italian primary school children [5], a lexical–semantic model was proposed, based on verbal input for writing numbers from dictation. The model includes a comprehension stage for numbers, which converts the perceived verbal number into semantic representation, followed by a production phase. They postulated that this semantic representation reflects the structure of the verbal number and the number’s basic components (e.g., units and decades) in an embedded number sum. This semantic representation then becomes the appropriate digit numeral in the production phase.

The two remaining models are asemantic. In contrast to semantic models, asemantic transcoding models do not require a magnitude representation when transcoding from one numerical notation to another. A model in which the transcoding of digit numbers into verbal numbers can occur through a direct route without the mediation of abstract representation was proposed [38]. The model distinguishes between four stages of processing. First, the analysis of the digit number starts from the right. Then, the process of categorization specifies the necessary parameters to implement the rules of transcoding, i.e., those related to the group and the position of the lexical numerical elements. Then, the transcoding process itself assumes the order of the numbers from the right place to fill the frame with slots with digits. Finally, at the production stage, a full number is stated.

An asemantic procedural transcoding model called ADAPT was proposed [39], wherein the transcoding of verbal numbers into digit numbers is initially performed with algorithmic strategies that are later replaced by the direct memory retrieval of numerical forms. When a person hears a verbal number, this string is stored in the database of phonological working memory. Then, the number string is divided into units so that each unit is the largest unit that is accessible in the long-term memory that fits its input. These units are subsequently processed sequentially through the production system. This transcoding process creates no semantic representation. Lexical errors are the result of difficulties in retrieving correct digit numbers from the long-term memory depending on the number of fetches that are required. By contrast, syntactic errors involve incorrect placement and the ordering of digits, e.g., reversal of tens and units.

### 1.3. Characteristics of the Number System in Arabic

Arabic uses a digit system for writing numbers: most commonly with Hindi rather than Arabic digits. Some Arab countries, such as the countries of the Near and the Middle East, use the Hindi system, while other countries such as Morocco use the Arabic system. The written numeral system (Arabic–Hindi digits: ٠١٢٣٤٥٦٧٨٩ and Arabic–Arabic digit numerals: 0123456789) is a variant of the base-ten number system used in most literate countries and considered to be highly effective for representing numbers [40,41,42] because it uses a single base dimension and the dimension of power. The base dimension is represented by the shape of the 10 digits, and the power dimension refers to the position of the digit in the number that affects its value [42]. The 10-numbers from 0 to 9 are easy to learn. For example, the number 4 can relate to the magnitude of “four” (4), “forty” (40), or “four hundred” (400). Understanding the position-value system in the digit number system is essential to engage with multi-digit numbers, and it is part of one representation of the extended version of the triple code model [43]. Therefore, the system of digit numbers not only has a small lexicon but also an effective and simple syntax, which together enable the representation of numbers both quickly and clearly and simplifies calculations.

According to the number taxonomy that was previously proposed [10,11], word numbers in Arabic include lexical elements that are arranged in groups of units, tens, hundreds, and multiples “مئة or مية” (one hundred) and “ ألف “ (thousand) to represent the values of a number by its syntax. In two-digit numbers, lexical elements are organized in a name-value system: a digit number receives its value according to its name rather than its position in the acoustic sequence. For example, the digit number 5, which is in the fifth place in different groups, is known as “خمسة” (five) as a unit and as “خمسين” (fifty) as a decade. In 15, it belongs to another group and is called “خمسة عشر—خمستعش” (fifteen). To represent all possible numbers, these elements are combined or multiplied by addition, e.g., “خمسة عشر (خمستعش)” (fifteen) corresponds to “ عشرة “ + ” خمسة “ (five + teen), or multiplication, e.g., “ مئة خمس (خمسمية)” (five hundred) corresponds to “ مئة “ × ” خمسة.” As with many other number systems, children who are beginning to count must memorize the words of one-digit numbers from one to nine and, subsequently, the number words for 10, 11, and 12. The word numbers for 13 to 19 can be derived from the one-digit word numbers, whereas the word numbers for 11 and 12 are not consistent, e.g., “11-أحد عشر (حداش\حدعش)” and-12-”إثنا عشر (تناش\تناعش)” but 1-”واحد” 2-”إثنين (تنين\ثنين)”. In sum, the tens system from 30 to 99 is regular, while from 11 to 29 peculiarities make the verbal numeral system less transparent. This inconsistency is probably due to the historical 12-based number system, and it tends to cause difficulties for young children who are learning to count numbers of tens. This is also a feature of other Middle Eastern and most European number systems, whereas Chinese and other East Asian number systems are more transparent from 10 onwards [44,45].

After learning the teens (11–19), Arabic-speaking children must also memorize the names of whole tens (20, 30, 40 …) that are identical or similar to one-digit word numbers adding the suffixes “ ين\ون “ at the end (6 = سته, 60 = ستة + ين = ستين\ ستة + ون = ستون). Moreover, the order of unit and decade word numbers in two-digit numbers is reversed in Arabic; for example, 18 is called “eight-ten” (“ثمانية عشر\ثمنتاش\ثمنتعش”) and 27 is called “سبعة وعشرين.” German word numbers have the same feature of inversion as Dutch, Danish, and sometimes Norwegian and Czech [24].

### 1.4. The Present Study

As discussed above, many linguistic differences have emerged for various numerical tasks, but only a few number-processing studies have engaged with the Arabic language [18,46]. The present study investigates the special Arabic numerical system, wherein numbers in general and two-digit numbers in particular differ syntactically from the numerical systems of most other languages. The syntactic structure of numbers in Arabic mainly differs in terms of the order of units and decades. In Arabic, two-digit numbers are read from right to left, i.e., the first digit is the units and the second is the decades (24 = four and twenty) but may be written from right to left, i.e., the first digit is the units and the second is the decades, or reverse from left to right, i.e., the first digit is the decades and the second is the units. Reversing the order of units and decades in Arabic is a basic, inherent feature for two-digit and multi-digit numbers [47,48]. Notably, in Arabic, this inverted order of units and decades (right to left) occurs in the same direction of the reading and writing of words (right to left). In other languages, such as Hebrew and German, the direction of reading words (Hebrew: right to left, German: Left to Right) and two-digit numbers is the opposite (Hebrew: Left to Right, German: right to left), and in English, they both occur from left to right. In a previous study it was claimed that the language and the reading direction of words and numbers in particular can affect the spatial mapping of the number line [48].

### 1.5. Objectives and Hypotheses

This study investigated the effect of the syntactic representation of numbers in Arabic on the task of transcoding two-digit numbers from dictation. For this purpose, it used the paradigm of writing two-digit numbers from dictation [2,3,5,7,9]. The study participants were primary, junior-high, and high school pupils and higher education students with Arabic as a mother tongue. They carried out a transcoding task, namely writing two-digit numbers from dictation. The two-digit numbers were in four categories: whole-tens numbers (e.g., 40, 50); teen numbers (e.g., 13, 15), identical-decade-unit numbers (e.g., 33, 44) and the remaining two-digit numbers (32, 61, 86, etc.).

Firstly, we predicted that, for at least some numbers, participants would use a units-first writing pattern for numerals, rather than the standard tens-first for at least some numbers. This has previously been found for the German language, which is another language with the inversion feature [22]; and was expected to be even stronger in Arabic because of the right-to-left writing direction for text in general and number words in particular. Secondly, we predicted that the syntactic complexity of the structure of the number words would influence the likelihood of participants using the decades-first versus the units-first writing direction. Thus, participants should have predominantly used the decades-first order for whole-tens numbers but may have used the units-first strategy for other numbers and were particularly likely to do so for the ‘remaining two-digit number’ category of numbers that are neither whole-tens numbers, teen numbers nor identical-decade-unit numbers.

Thirdly, we predicted a developmental shift. such that older participants would be increasingly more consistent in using decades-first writing strategies, and in particular, that primary school pupils would be more likely to use units-first strategies, especially for numbers in the ‘remaining two-digit number’ category, than those in junior high school, high school, and higher education. This is both due to the fact that, with increasing exposure to number reading and writing as people progress through education, the standard decades-first writing strategy is likely to become more automatized, and because working memory increases with age. It is likely that the decades-first writing strategy demands place a significant load on working memory, due both to the inconsistency between expected number writing and text writing patterns, and to the inversion feature. Therefore, younger participants with lower working memory are likely to find it more difficult to use and may therefore be more likely than older participants to adopt the units-first strategy.

We also hope that the results may contribute to assessing the validity of different models, summarized above, and listed in Table 1. The table gives the predicted writing order for writing decades and units, according to the different models. The task here may not in fact be highly sensitive to most differences between the models, as most models predict similar results: however, it discriminates successfully between the abstract modular model and the rest.

Most of the models predict a decades-first strategy for whole-tens words and a units-first strategy for the other categories. This was expected to be the case for the triple-code model, since the transcoding process occurs in a direct route according to the phonological activation. with no need for a semantic representation, for the encoding complex model, and IR model, since in both the latter cases the transcoding process depends on the specific (verbal or numerical) codes of the format and modality Power and Dal Martello’s model predicts the same, despite being format-independent, since the transcoding process occurs within a semantic representation that reflects the structure of the verbal number and its basic components (e.g., units and decades). The same is true of Deloche and Seron’s model, since the transcoding process occurs through a direct route with no need for a semantic representation and of the ADAPT model, since the transcoding process depends on the accessibility of word numbers and digit numbers in long-term memory. The one model that would predict a consistent decades-first writing pattern is McCloskey’s abstract modular model since it is independent of the number format (verbal, numeric) and the semantic representation of numbers is created from left to right according to the exponent of 10.

## 2. Materials and Methods

Participants: There were 287 participants in total. They included 77 pupils (56 male, 21 female) from primary school, 66 pupils (52 male, 14 female) from junior high school, 72 pupils (45 male, 27 female) from high school and 72 students (38 male, 34 female) from higher education. The primary school children included four children from grade 1, 11 from grade 2, 12 from grade 3, 18 from grade 4, 23 from grade 5 and nine from grade 6. The junior high pupils included 16 from grade 7, 28 from grade 8 and 22 from grade 9. The high school pupils included 24 from grade 10, 18 from grade 11 and 30 from grade 12. In the Israeli school system, school begins at age 6, so grade 1 pupils are aged 6 to 7, grade 2 pupils aged 7 to 8, and so on until grade 12, where pupils are aged 17 to 18.

All participants lived in Arab-majority areas of Israel and spoke Arabic as a mother tongue (L1). According to teachers’ and parents’ reports, none of them suffered from specific difficulties in mathematics or other academic issues.

Task: The transcoding tasks consisted of the writing of digit numbers pre-recorded by the experimenter in Palestinian Arabic. Participants transcoded two-digit numbers from dictation. Numbers ranged from 12 to 99 100 and included 8 numbers from each of the four two-digit number categories: (1) Teen-numbers: 12-13-14-15-16-17-18-19, (2) Identical-units-decade numbers: 22-33-44-55-66-77-88-99, (3) Whole-tens numbers-20-30-40-50-60-70-80-90 (10 was excluded because the verbal word of ten in Arabic عشرة does not have the suffix ون\ين as the other whole tens) and (4) The remaining two-digit numbers: 26-37-48-59-61-72-83-94. The order in which numbers were presented was random for each participant.

Procedure: The experimenter tested each participant individually in a quiet room. Participants and the experimenter were seated at a table so that participants could not read the comments that were written about their performance. Participants did not receive any feedback about their answers. They could request a short break during the test if they desired one.

For the transcoding task, participants were asked to write digit numbers from dictation. In this task, participants were asked to write numbers from dictation on a blank sheet of white paper (A4). A pre-recorded experimenter dictated in Palestinian Arabic one two-digit number at a time to the participants. If necessary, e.g., if the participant did not hear the number, the experimenter replayed the dictated number again. During the number writing task, the experimenter noticed whether the participant wrote the units first or decades first.

## 3. Results

The units first and decades first writing pattern rates were calculated according to the four number categories—teen numbers, identical-unit-decade numbers, whole-tens numbers and the remaining two-digit numbers—as a function of educational level -primary, junior high, high, and higher education. A mixed-repeated measures analysis of variance (Mixed-RM-ANOVA) was conducted for the units first and decades first writing pattern rates, with number category as the within-subject variable and educational level as the between-subject variable.

**Figure 1:**
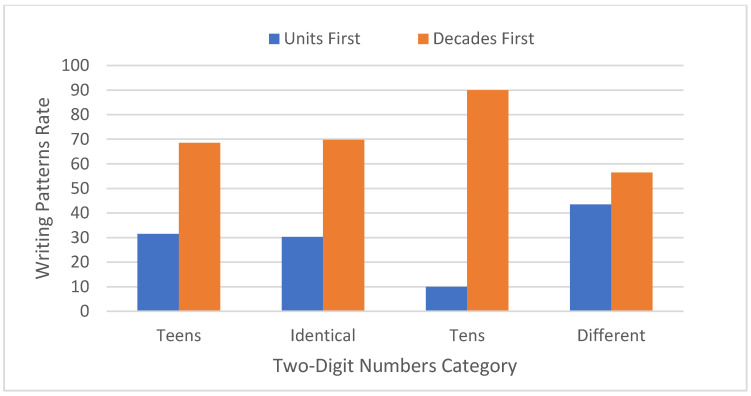
The units first (right to left) and decades first (left to right) writing pattern rates as a function of two-digit numbers category (teens, identical, tens and different).

A significant effect of number category was observed (F [3,849] = 46.85, MSE = 5.47, *p* < 0.0001). In a post hoc analysis of paired comparisons for the units first (right to left) writing pattern rate, this pattern was more frequent for the remaining two-digit numbers than for teen numbers or identical decade-unit numbers, which did not differ significantly from one another in this respect, and in turn showed a higher units-first writing pattern rate than whole-tens two-digit numbers that elicited the lowest number of units-first responses (see Figure 1).

**Figure 2:**
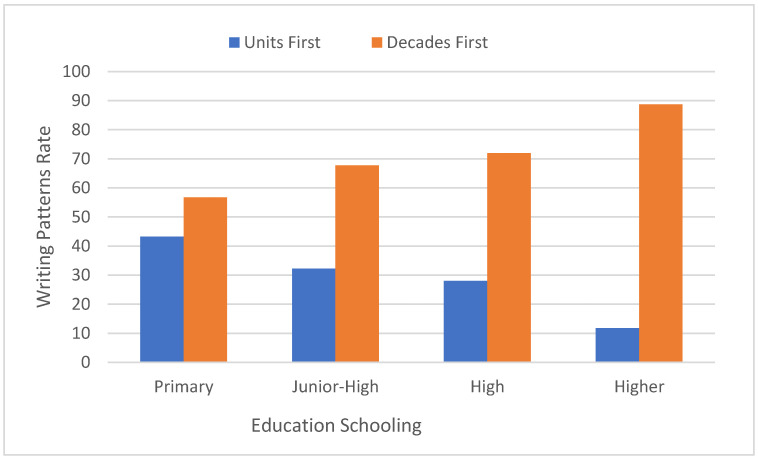
The units first (right to left) and decades first (left to right) writing pattern rates as a function of educational level (primary, junior-high, high, higher).

There was a significant main effect of educational level:-(F [3,283] = 262.4, MSE = 95.74, *p* < 0.0001). In a post hoc analysis of paired comparisons of the units first (right to left) writing pattern rate was higher for primary school pupils than for junior-high or high school pupils, who did not differ from one another, and showed higher rates than that for higher education students (see Figure 2).

**Figure 3:**
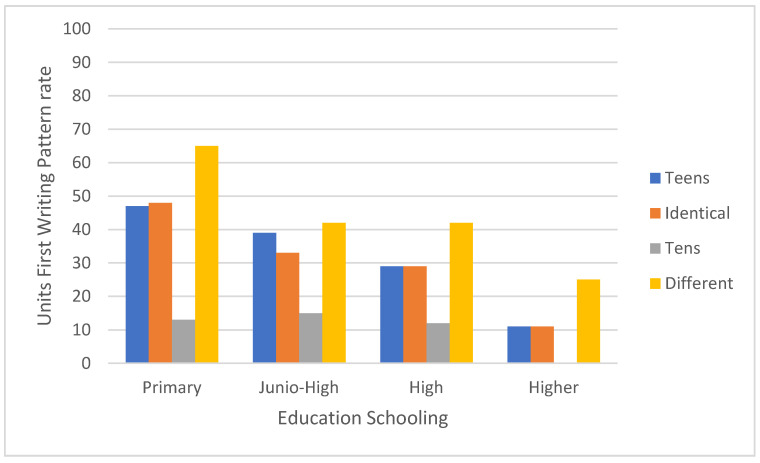
The units first (right to left) writing pattern rates as a function of two-digit numbers category (teens, identical, tens and different) and educational level (primary, junior-high, high, higher).

**Figure 4:**
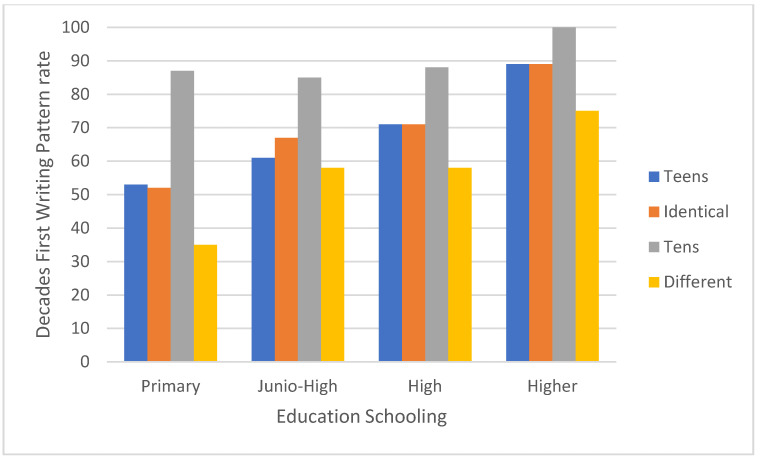
The decades first (left to right) writing pattern rates as a function of two-digit numbers category (teens, identical, tens and different) and educational level (primary, junior-high, high, higher).

Figure 3 and Figure 4 show a significant interaction between educational level—primary versus junior high versus high school versus higher education, and number category—teen numbers versus identical unit–decade numbers versus whole-tens numbers versus the remaining two-digit numbers (F [9,849] = 2.23, MSE = 0.26, *p* < 0.05). In a post hoc analysis of paired comparisons of the units first (right to left) writing pattern rates in primary school pupils: the rate was higher for the remaining two-digit numbers than for teen numbers or identical-units-decade numbers, which did not differ significantly from one another, and showed higher rates than for whole-tens numbers (see Figure 3 and Figure 4). Similar findings were obtained for higher education students. For both junior high and high school pupils, the units-first writing pattern rate did not differ significantly between teen numbers, identical-unit-decade numbers, and the remaining two-digit numbers; but was higher for each of these categories than for whole-tens numbers.

## 4. Discussion

The current findings suggest that, in general, Arabic speakers adopt the decades-first writing pattern in the transcoding of two-digit numbers. They used the decades-first writing pattern for 78% of items and the units-first writing pattern for only 22% of items. Thus, our first prediction was only partially supported. The participants did use the units-first pattern for a significant minority of items, which probably would not occur in most other languages for participants beyond the first year or two in primary school, though a direct comparison of different language groups on the same task would be desirable in future research. However, they used the decades-first pattern much more frequently,

This pattern is in line with a recent study [49] which showed that native speakers of Arabic performed better in a digit writing task requiring the transcoding of number-words into numerals when the numbers were presented in the non-inverted format (HDU), a format that is nonstandard in Arabic, compared to transcoding numbers presented in the inverted standard format of Arabic (HUD). In addition, when the stimuli were presented (in both modalities) in the HDU format, speakers of Arabic made very few changes in the writing direction, writing the digits in the same order that they heard or saw them. However, when the number words were presented in the standard, inverted, format in Arabic (with units and decades reversed, HUD), only 14/46 participants consistently (in 95% or more of the trials) wrote the numbers in the order of hundreds, decades then units, in one or both modalities. Most speakers of Arabic tended (in 3 or more of the 23 trials of the test block) to write the hundreds, leave a space, write the units, and then enter the decades into the space. This form of writing multi-digit numbers was never observed in by native speakers of Hebrew (which lacks the inversion feature), even when presented with number words in the reverse-order HUD format (either in Hebrew or in English).

Our second prediction was supported: number category—teen numbers, whole-tens numbers, identical-unit-decade numbers, or the remaining two-digit numbers—affected the direction of number writing. Units first (right to left) writing pattern rates were higher for the remaining two-digit number category than for teen numbers or identical unit–decade numbers, which were in turn higher than for whole-tens numbers. Thus, that the syntactic structure of the numerical system (order of units and decades) of Arabic language, especially of whole tens (decades first- 6 = سته, 60= ستة + ين = ستين\ ستة + ون = ستون), teens (where the units of this category are specific for it as previously described) and remaining different two-digit numbers (units first- ستة واربعون = 6 + 40) affects the transcoding process in the two-digit number writing from dictation task differentially. Since whole-tens numbers are dictated with decades first, even primary school pupils adopt the decades-first writing pattern (left to right) as in standard pattern for number writing but in the opposite direction to text writing (right to left) in Arabic. For the remaining two-digit number category, primary school pupils adopted the units first writing pattern (Right to Left) as in text writing but unlike the standard number-writing direction. Though older participants made predominant use of the decades-first strategy even for the remaining two-digit number category, junior-high pupils, high school pupils and even higher education students used the units-first writing pattern more frequently for this category than for the other categories.

There is a similarity between the performance of the participants in the current study and Arabic-Hebrew bilingual adults of a recent study [50]. In this recent study, the paradigm of reading and writing two-digit numbers from dictation in both languages was used. Sixty university bilingual students were given two tasks in both Arabic and Hebrew: One task involved writing two-digit numbers to dictation, and the other involved reading two-digit numbers aloud. Reading times and the error rates were calculated in both languages according to type of error—total errors, substitution errors, change errors, and omission errors. The participants made some errors in reading, especially in writing two-digit numbers. Their most common errors were substitution errors compared to change and omission errors. Such errors were more common for numbers which require processing the numerical syntactic structure than for decade numbers, or numbers from 11 to 19, which require less attention to numerical syntax. The same was found in another recent study [51] with first graders, suggesting particular difficulty with the syntactic rather than lexical aspects of the counting system. The syntactic aspects may be particularly difficult for Arabic-speaking children due to the inversion feature of the Arabic counting system.

Our third prediction was also supported: there were differences between participants of different ages and educational levels. The rate of use of the units-first (right to left) writing pattern was highest for primary school pupils, who actually used it for a majority of items in the remaining two-digit number’ category. Junior high and high-school pupils were similar to one another with regard to the rate of use of units-first and decades-first writing patterns, while higher education students were the most consistent in their use of the decades-first pattern and least likely to use the units-first pattern. It appeared that the older pupils and the higher education students were less readily influenced in their number writing by the syntactic structure of the particular numbers dictated, because of both greater mathematical proficiency and greater flexibility in separating oral and written number patterns as a result of experience with a second language (Hebrew) and a third language (English), with different directions of number and text writing and different syntactic structures for number words. In Hebrew, as in Arabic, text writing is from right to left and the standard number writing pattern is from left to right, but, unlike Arabic, the inversion feature is not present, and decades precede units in number words (except teen words). In English, unlike both Hebrew and Arabic, text writing is from left to right; as with both the other languages, the standard number writing pattern is from left to right; and, as in Hebrew but not Arabic, the inversion feature is not present, and decades precede units in number word (except teen words). This exposure to several different number representation systems may reduce the rigidity of associations between the syntax of number words and numeral combinations. Additionally, improvements in working memory may lead to greater proficiency, not only in the ability to remember the lexical elements and their sequence, but also through the ability to manipulate the sequence of digits in multiple formats and verbal notations [24,52,53]. It has been proposed that in a transcoding task, when decades come after units and the order needs to be reversed, there are larger demands on working memory processes [39].

Junior high school pupils, high school pupils and higher education students are more proficient than primary school pupils in mathematics in their second language of Hebrew (where text is written from right to left, numbers are written from left to right, and there is a decades-units syntactic structure) and in their third language of English (where text is written from-left to right, numbers are written from left to right, and there is a decades-units syntactic structure). By this stage, they depend less on the syntactic structure of two-digit numbers (the order of units and decades) induced by dictation and are thus more likely to use decade-first writing patterns for all numbers. Thus, their writing patterns at this stage are consistent with McCloskey’s abstract modular model, and inconsistent with the other models. These patterns require them not only to remember the lexical elements and their sequence, but also to manipulate the sequence of digits in multiple formats and verbal notations [24,52,53]. It has been proposed that such transcoding tasks, where decades come after units and the order needs to be reversed, place a greater load on working memory [39]. Thus, the move with age to decades-first writing patterns may involve not only greater mathematical and linguistic expertise, but improvements in working memory. Further studies should investigate the relationships between unit- versus decade-first number-writing patterns and performance on working memory tasks; and also look at whether performance on transcoding tasks is influenced by interference with phonological working memory, for example through articulatory suppression [54].

An interaction was observed between educational level -primary, junior high, high school or higher education and number category—teen numbers, whole-tens numbers, identical-unit-decade numbers. and the remaining two-digit numbers. In primary school children, the units first (right to left) writing pattern rate was higher than for the remaining two-digit number category than for teen numbers or identical unit-decade numbers and was higher for both than for whole-tens numbers. This was also true of the higher education students, though their overall rates of usage of the units-first strategy were much lower. For both junior high and high school pupils, the rate of use of the units-first strategy was higher for numbers in the remaining two-digit number category than for any of the other categories, which did not differ from one another.

One possible limitation of the study with regard to effects of differences in educational level is that the educational levels, especially primary school, were quite broadly defined and included a fairly wide age range within each level. Future studies should include a finer-grained investigation of primary school children’s progress from age 6 to 12.

The results support the findings for European languages that indicate that transcoding is affected not only by general factors but also linguistic ones [23,24]. The findings of the present study clearly indicate that the complicated linguistic structure of two-digit numbers in Arabic and its inversion feature affect the transcoding of numbers from one notational format to another [21].

The differential influence of the syntactic structure on the units first\decades first writing pattern in the transcoding task (writing from dictation) is consistent with most of the proposed models, only ruling out the abstract modular model, which would predict a consistent decades-first response. More research needs to be carried out to compare the validity of the other models for different number systems.

## 5. Conclusions

To summarize, the findings of the current study indicate that, in general, Arabic speakers adopt a decades-first writing pattern for two-digit numbers. especially when this is consistent with the syntactic structure of two-digit numbers. as in whole-tens numbers. This decades-first writing pattern becomes more consistent as students move beyond primary school to junior-high school, high school, and higher education. However, this pattern is modulated by the complexity of the numerical syntactic structure and, as we propose, by the working memory capacity that it requires. This complexity is less marked in whole-tens numbers, teen numbers and identical-decade-unit numbers than in the remaining two-digit numbers. The writing pattern in transcoding also seems to be influenced by the level of consistency between the reading direction for text, the reading direction for two-digit numbers and the taught direction for writing two-digit numbers.

These findings have some implications for mathematics education. They imply that educators should give attention to children’s mastery of the transcoding of verbal word two-digit numbers into Arabic digits, especially to their writing patterns for two-digit numbers (decades-first or units-first). This is especially true of languages with counting systems with the unit–decade inversion feature. The clear impact of the unit–decade inversion feature of two-digit number transcoding has implications for the planning of future mathematics curricula and textbooks.

The initial stage of teaching transcoding to Arabic-speaking children, and possibly other speakers of languages with inverted counting systems, should begin with the whole-tens numbers, which elicit the decades-first writing pattern and require mainly just short-term memory rather than working memory. Pupils should then move to teen numbers and identical unit–decade numbers and then to the remaining two-digit numbers, since the latter numbers make the greatest demands on working memory and on processing the numerical syntactic structure. We conclude that an earlier focus on two-digit number transcoding (starting. in first and second grade) may have positive effects on the development of numeracy since it would give children an earlier start in grasping the difficult syntactic structure of two-digit numbers and might thereby improve their ability to deal with more complex mathematical tasks involving two-digit and multi-digit numbers.

## Figures and Tables

**Table 1:** Summary of number processing models and their predicted units\decades first writing patterns in two-digit numbers transcoding to dictation.

Number Processing Model	Number Representations, Formats, Codes, Elements	Semantic Representation	Format Dependent (Verbal, Numeric)	Transcoding Process	Expected Units First\Decades First Writing Patterns
**The Triple Code**	Auditory-Verbal	No	No	Direct	Units First
	Numeric-Verbal				
	Analog				
**Deloche and Seron**	Analysis	No	Yes	Direct	Units First
	Categorization				
	Implementation				
	Production				
**ADAPT**	Memory	No	Yes	Direct	Units First
**Abstract Modular**	Comprehension	Yes	No	Abstract representation	Decades First
	Production				

**The Encoding Complex**	Verbal	No	Yes	Reading dependent	Units First
	Non-verbal				
**Intermediate Representations-IR**	Lexical	Yes	Yes	Intermediate representation dependent	Units First
	Syntactic				
**Power and Dal Martello**	Comprehension	Yes	No	Lexical-semantic representation	Units First
	Production				

## Data Availability

The data presented in this study are available on request by the corresponding author.
